# Colonization by arbuscular mycorrhizal fungi improves salinity tolerance of eucalyptus (*Eucalyptus camaldulensis*) seedlings

**DOI:** 10.1038/s41598-021-84002-5

**Published:** 2021-02-23

**Authors:** Chaiya Klinsukon, Saisamorn Lumyong, Thomas W. Kuyper, Sophon Boonlue

**Affiliations:** 1 Department of Microbiology, Faculty of Science, Khon Kaen University, Khon Kaen, 40002 Thailand; 2 Department of Biology, Faculty of Science, Chiang Mai University, Chiang Mai, 50200 Thailand; 3 Soil Biology Group, Wageningen University & Research, P.O. Box 47, 6700 AA Wageningen, The Netherlands; 4 Research Center of Microbial Diversity and Sustainable Utilization, Chiang Mai University, Chiang Mai, 50200 Thailand; 5 Academy of Science, The Royal Society of Thailand, Bangkok, 10300 Thailand

**Keywords:** Microbiology, Plant sciences

## Abstract

Soil salinity affects soil quality and reduces plant performance. Arbuscular mycorrhizal fungi (AMF) can enhance the tolerance of plants under salinity stress. Cultivation of eucalyptus (*Eucalyptus camaldulensis*), which exhibits high water use efficiency, is possible in saline areas to produce raw materials for the pulp industry. We determined the effects of arbuscular mycorrhizal fungi (AMF) on the growth and survival of eucalyptus seedlings under saline conditions. Three different clones of eucalyptus seedlings were pre-inoculated with three salt-tolerant AMF species, namely *Glomus* sp.2, *Gigaspora albida* and *G. decipiens*, and without pre-inoculation. The seedlings were grown in a greenhouse for 45 days. They were then transferred to individual pots, filled with field soil and subsequently treated with NaCl solution until electro-conductivity (EC) reached 10, 15 and 20 dS m^−1^. They were watered for 90 days under nursery conditions. The results show that increased salinity levels reduced plant performance, fractional AMF root colonization, spore number, and eucalypt K/Na ratio. AMF significantly increased chlorophyll and decreased leaf proline concentrations by more than 50% and 20% respectively and increased the K/Na ratio three- to six-fold compared with non-inoculated plants. Pre-inoculation with AMF before outplanting also improved plant performance by more than 30% under salinity stress compared to non-inoculated plants. We conclude that AMF can alleviate the negative impacts of salinity on plant physiological and biochemical parameters.

## Introduction

Saline soils in the twenty-first century are increasing^1^. Increased salinization of arable land may have a large negative global effect, predicted to result in a 30% loss of land within the next 25 years, and up to 50% by the middle of the twenty-first century^2^. Soil salinity is a serious problem for agriculture, particularly in arid and semi-arid regions^3^. Salinity limits plant growth and crop productivity. Under salinity conditions, the three main problems for plant growth include osmotic stress (physiological drought), the toxic effect of ions, notably sodium (Na) ions, and nutrient imbalance^4^. Arbuscular Mycorrhizal Fungi (AMF) form symbiotic associations with the roots of many plant species. AMF species occur naturally in saline environments^5^. Recent studies have highlighted the benefits of AMF for host plants by improving soil quality, enhancing growth, regulating substances, and resistance to plant pathogens and environmental stress^6–9^. Through selective ion uptake they can improve the ionic balance, as expressed in the ratio between K^+^ and Na^+^ (K/Na ratio)^10^. Furthermore, AMF can increase enzyme activities, protect enzymes from damage, and enhance antioxidant production. Under salinity stress, mycorrhizal plants grow better than non-AMF plants due to increased nutrient uptake, photosynthesis, water use efficiency, the production of osmoprotectants, higher K/Na ratios, and compartmentalization of Na within certain plant tissues^11^. These beneficial effects of AMF depend on the behavior of individual fungal species and strains^12^. Eucalyptus or river red gum (*Eucalyptus camaldulensis* Dehnh.) is a fast-growing plant native to Australia. The species can grow in a wide range of soils, from very poor to rich soils. It is one of the economically most important trees in Thailand, being used as raw material in the production of pulp, oil, furniture and housing^13^. Three eucalyptus species; *E. alba* Blume (white gum), *E.microtheca* F. Muell. (coolibah), and *E. camaldulensis* have been investigated earlier for their salt tolerance^14^. *Eucalyptus camaldulensis* is the first choice for many growers in Thailand as it can adapt to the saline soils in the northeastern region of Thailand, such as in Khon Kaen and Kalasin province, where soils range from slightly to strongly saline (EC 4–16 dS m^−1^)^15^. The species is also tolerant to various climate conditions. Cultivation of this species is therefore one option for producing wood in areas with saline soils. An effective way of expanding saline-land usage in Thailand could be the use of saline-tolerant strains of AMF, isolated from the eucalyptus rhizosphere in saline soils, to improve plant tolerance. This study therefore aimed to investigate the contribution of AMF to survival and growth of eucalyptus seedlings under salinity stress. We executed a three-factorial experiment with saline-tolerant strains of AMF, different eucalyptus clones (C), and soil salinity levels (S).

## Materials and methods

### AMF inoculum and plant preparation

Three AMF species, which are most frequent in saline soil areas in Khon Kaen province, viz., *Glomus* sp.2 (KKU-BH-001), *Gigaspora albida* (KKU-BP-001) and *G. decipiens* (KKU-BP-002), were isolated from the rhizosphere of eucalyptus from planting sites on saline soil in Ban Phai (EC 6.92 dS m^-1^) and Ban Haeat district (EC 5.35 dS m^−1^). These AMF isolates were selected after screening their salt tolerance by growing them in soils supplemented with a strong NaCl solution (EC 20 dS m^−1^) using a minor modification of the sandwich technique^16^. Briefly, AMF spores (20–30 spores) were surface-sterilized by 2% chloramine-T and washed with sterilized distilled water 4–5 times. Thereafter, surface-sterilized AMF spores were placed between two sheets of gridline sterile filter membranes with pore size diameter of 45 μm and covered by plastic frame slides that are called Hepper units. Hepper units were embedded in Petri dishes containing sterilized saline soil. Soils in each dish were watered with 25 mL sterilized distilled water and incubated in the dark at room temperature for 15–20 days. After incubation, spore germination was checked by removing the Hepper unit from the plate, cleaning by tap water, and staining with acetic glycerin solution with trypan blue. After staining, the filter membrane was gradually separated, and spore germination was observed under a stereomicroscope (Nikon SMZ445). The AMF species were subsequently propagated in maize (*Zea mays* L.) by the pot culture technique in a sterilized sandy loam. Pots were then placed in a greenhouse under natural lighting conditions for three months. Colonized root fragments (fractional root colonization 70–90%) and spores (24 spores g^−1^ dry soil) were used as inoculum. Forty-five days old eucalyptus cuttings from three clones that differ in salt tolerance were used: commercial clone H4, which can grow in sand, clone P6, which can grow in loam, and non-commercial clone H8, which can grow in sandy loam. Cuttings were obtained by using the patent of SCG packaging public company limited, Phoenix Pulp & Paper Public Co. Ltd. and Siam Forestry Co., Ltd, Thailand. Cuttings were grown in sterilized coconut dust and subsequently inoculated with 40 g inoculum in the mycorrhizal treatments, and 40 g sterilized inoculum in the non-mycorrhizal treatment.

### Experimental design

The eucalyptus cuttings were transplanted into individual pots that were filled with 20 kg field soil, with the following properties: pH 4.87, EC 5.72 dS m^−1^, soil organic matter 3.5 g kg^−1^, total N 195 mg kg^−1^, total P 50 mg kg^−1^, total K 5,950 mg kg^−1^, exchangeable Ca 100 mg kg^−1^ and Na 464 mg kg^−1^. The experiment was a 3 × 3 × 4 complete factorial experiment in a randomized complete block design (RCBD) with three salinity levels (10, 15 and 20 dS m^−1^), three eucalyptus clones (H4, H8 and P6) and four AMF treatments (*Glomus* sp.2 KKU-BH-001, *G. albida* KKU-BP-001, *G. decipiens* KKU-BP-002, and a treatment without AMF pre-inoculation). Each treatment had three replicates. After fourteen days, to avoid plant shock from salinity, 5% of NaCl solution was gradually added to the soil every seven days to increase the initial EC from 5.72 (0% NaCl) to 10, 15 and 20 dS m^−1^, respectively. All eucalyptus cuttings were watered with 1,000 mL distilled water every three days, and excess water in saucer was reused in order to maintain salinity. Every six days before watering the pots we took soil samples to check the EC. Assessment of plant and fungal performance parameters was conducted at 90 days.

### Assessment of plant and fungal parameters

Plant fresh and dry weight (g), and plant height (cm) were measured. Eucalyptus roots were scanned by an Epson scanner V700 PHOTO and analyzed with WINRHIZO Pro2004a (REGENT Instruments Inc., Qc, Canada). We assessed root length and root diameter and calculated on that basis specific root length, root surface area, and root tissue density.

Mycorrhizal root colonization was determined after staining with acetic glycerin solution with trypan blue and scoring root fragments with the method proposed by Trouvelot et al.^17,18^. Spore density (number of spores g^−1^ dry soil) was observed after sucrose centrifugation^19^.

Intensity of AMF colonization (I) was calculated using the following equation:

$${\text{I}}\left( \% \right) = \left( {\left( {{\text{95n5}} + {7}0{\text{n4}} + {3}0{\text{n3}} + {\text{5n2}} + {\text{n1}}} \right)} \right){\text{/N}}$$

where “n5” means AMF root colonization level 5 (90–100%), “n4” is level 4 (50–90%), “n3” is level 3 (10–50%), “n2” is level 2 (1–10%), “n1” is level 1 (0–1%) and N is the total number of root segments.

### Plant nutrient analysis

Plant N concentration was determined after digestion by the Kjeldahl method and analyzed by the FLA method^20^, while plant P and K concentrations were determined by the wet oxidation method^21^ and Na concentration determined by flame photometer^22^.

### Leaf relative water content (LRWC)

Leaf disc samples (10 mm diameter) were punched from each plant after 90 days to determine the tolerance of mycorrhizal and non-mycorrhizal plants at each salinity level. We calculated LRWC using the following equation^23^:

$${\text{LRWC}} \left( \% \right) = \frac{{\text{FW - DW}}}{{\text{TW - DW}}} \times 100$$

where FW is leaf fresh weight, DW is leaf dry weight after 24 h of drying at 70 °C, and TW is leaf turgid weight after being soaked in distilled water for 24 h.

### Leaf chlorophyll concentration

Leaf chlorophyll concentration (chlorophyll a, chlorophyll b, and total chlorophyll) was determined by the method described by Arnon^24^. Fresh leaves (0.5 g) were ground with 20 mL of 80% acetone. The homogenate was then centrifuged at 4,000 rpm for 15 min. The supernatant was read using a spectrophotometer (Thermo Scientific GENESYS 10S UV/Vis Spectrophotometer, model EW-02654–22) at absorbance readings at 645 (A645) and 663 (A663) nm. The chlorophyll content was calculated using the following formulae:

$$\begin{aligned} & {\text{Chlorophyll}}\;{\text{a}}\;\left( {{\text{mg}}\;{\text{gFW}}^{{ - {1}}} } \right) = \left( {{12}.{7} \times {\text{A663}}} \right){-}\left( {{2}.{69} \times {\text{A645}}} \right) \\ & {\text{Chlorophyll}}\;{\text{b}}\;\left( {{\text{mg}}\;{\text{gFW}}^{{ - {1}}} } \right) = \left( {{22}.{9} \times {\text{A645}}} \right){-}\left( {{4}.{67} \times {\text{A663}}} \right) \\ & {\text{Total}}\;{\text{chlorophyll}}\;\left( {{\text{mg}}\;{\text{gFW}}^{{ - {1}}} } \right) = \left( {{8}.0{2} \times {\text{A663}}} \right) + \left( {{2}0.0{2} \times {\text{A645}}} \right) \\ \end{aligned}$$

### Proline concentration

Proline concentrations were determined using the method described by Bates et al.^25^. Fresh leaves (0.5 g) were homogenized in 10 mL of 3% sulfosalicylic acid and then sieved through Whatman’s No. 1 filter paper. Then 2 mL filtrate solution were mixed with 2 mL of acid-ninhydrin and glacial acetic acid in a test tube, respectively. The reaction mixture test tubes were placed in a water bath at 100 °C for 1 h and then placed in ice to stop the reaction. The mixture was extracted by 4 mL toluene and the chromophore containing the toluene was separated to measure absorbance of 520 nm using a Thermo Scientific GENESYS 10S UV/Vis Spectrophotometer (model EW-02654–22). The calculated proline concentration was then compared with the proline standard.

### Statistical analysis

The treatment effects and the interactions were tested by three-way analysis of variance (ANOVA) using the Statistix program version 8.0. All data complied with the ANOVA assumptions of homoscedasticity and normality. Means were compared between treatments using Tukey's Honestly Significant Difference (HSD) at a 0.05 probability level.

## Results and discussion

Results of the analysis of variance are provided in Table 1. In almost all cases, salinity and AMF were significant sources of variation. Interactions between AMF and clone were significant sources of variation (except for leaf P concentration), indicating species-specific AMF responses on different eucalyptus clones. Eucalyptus clone and the other interactions were significant sources of variation for a number of parameters as well.

**Table 1:** ANOVA table showing the effects of arbuscular mycorrhizal fungi (AMF), salinity (S) and *Eucalyptus* clone (C) and their interactions on mycorrhizal fungal and plant traits.

	AMF	S	C	S × C	AMF × S	AMF × C	AMF × S × C
AMF colonization	**	**	**	ns	**	**	ns
AMF spore density	**	**	ns	ns	**	**	ns
Shoot fresh weight	**	**	ns	ns	ns	**	ns
Shoot dry weight	**	**	ns	ns	ns	**	ns
Root fresh weight	**	**	**	ns	ns	**	*
Root dry weight	**	**	**	ns	ns	**	ns
Root length	**	**	ns	ns	ns	**	ns
Root surface	**	**	ns	**	ns	**	ns
Leaf relative water content (LRWC)	**	**	**	**	ns	**	ns
Chlorophyll a	**	**	**	**	**	**	**
Chlorophyll b	**	**	**	**	**	**	**
Total chlorophyll	**	**	**	**	**	**	**
Proline	**	**	**	**	**	**	**
Leaf Nitrogen (N)	**	**	**	*	**	**	**
Leaf Phosphorus (P)	**	**	*	ns	ns	ns	ns
Leaf Potassium (K)	**	**	**	ns	ns	*	ns
Leaf Sodium (Na)	**	**	**	**	**	**	**
Leaf K/Na ratio	**	**	**	**	**	**	**

*Ns* non-significant, *Significant at *P* ≤ 0.05 and **Significant at *P* ≤ 0.01.

### AMF colonization and spore density

Control plants (plants that were not pre-inoculated) were also colonized by AMF, which was caused by the experiment, which, after pre-inoculation or not in sterilized soil, was executed in non-sterile field soil, however, colonization levels were much lower than in the pre-inoculated seedlings. Spore density and fractional root colonization significantly (*P* ≤ 0.05) declined with increasing salinity levels (Table 2). Mycorrhizal colonization and spore density were very significantly correlated (r = 0.64; n = 36; *P* < 0.001). The significant interaction between AMF and salinity level for both parameters (Table 1) indicated that the protective effect of pre-inoculation diminished at higher salinity levels. The interaction between AMF and eucalyptus clone was also significant for fractional root colonization, suggesting species-specific responses to different eucalyptus clones. Root colonization and spore densities with clone H4 and H8 were highest with *G. albida*, while eucalyptus clone P6 showed highest spore densities and root colonization with *Glomus* sp.2. AMF are generally characterized as showing little or no host specificity, however plant species or plant variety-specific responses to individual species of AMF have been observed before^26,27^. Our results are consistent with earlier studies that showed that salinity inhibited spore germination, suppressed the growth of hyphae after initial infection, and reduced the number of arbuscules^28–31^***.***

**Table 2:** Effect of salinity on AMF spore density (spore number g^−1^ dry soil; SD) and intensity of root colonization (I) of three eucalyptus clones (H4, H8, P6) pre-inoculated with various species of AMF after 90 days of cultivation at three salinity levels. AMF1; *Glomus* sp.2, AMF2; *G. albida*, AMF3; *G. decipiens*, and control (C); without pre-inoculation.

Treatments	10 dS m^−1^		15 dS m^−1^		20 dS m^−1^	
	SD	I (%)	SD	I (%)	SD	I (%)
H4 (C)	2.8b	38b	1.3b	24b	1.1b	13c
H4 + AMF1	5.5a	84a	3.8a	78a	1.9ab	65ab
H4 + AMF2	5.5a	86a	3.8a	82a	2.7a	74a
H4 + AMF3	5.6a	81a	3.0a	68a	2.7a	63b
H8 (C)	1.7b	22c	0.8b	19b	1.0b	11c
H8 + AMF1	5.0a	83b	3.3a	76a	1.2b	64b
H8 + AM2	6.2a	92a	5.0a	84a	2.9a	71a
H8 + AM3	4.1ab	85b	3.2a	81b	1.6b	66ab
P6 (C)	1.6b	25b	1.2c	12c	1.1b	10c
P6 + AMF1	7.4a	90a	5.0a	80a	2.6a	70a
P6 + AMF2	3.9b	84a	2.9b	69b	1.5b	51b
P6 + AMF3	3.9b	81a	2.5bc	63b	1.6ab	54b

Values followed by different letters, per salinity level and clone, are significantly different (*P* ≤ 0.05) by HSD.

### Plant performance

Both AMF and salinity were significant sources of variation for root and shoot biomass, whereas clone was only a significant source of variation for root parameters. The interaction between AMF and clone was also significant, again demonstrating AMF species-specific responses in combination with different clones (Table 1). Salinity decreased plant performance parameters, with a larger effect at higher salinity levels, whereas pre-inoculated plants produced more biomass than control plants. At all salinity levels, plants pre-inoculated with *G. albida* usually showed higher biomass than plants pre-inoculated with the other AMF species (Table 3). However, at the salinity level of 15 dS m^−1^, eucalyptus clone P6 pre-inoculated with *Glomus* sp.2, was significantly heavier than when pre-inoculated with the other AMF species. These data fit with the selectivity of the different AMF for different clones as assessed by fractional root colonization and spore density. Negative effects of salinity have been reported for many glycophytes, such as *Allium cepa* L., *Medicago sativa* L., *Triticum aestivum* L. and *Hordeum vulgare* L.^28,29,32^ and the alleviation of these negative effects of salinity by AMF, and plant and fungal species specificity with respect to this protective effect has also regularly been reported^31,33–35^.

**Table 3:** Influence of different salinity levels on leaf relative water content (LRWC), root fresh weight (RFW), root dry weight (RDW), shoot fresh weight (SFW), shoot dry weight (SDW), plant height (PH), root length (RL), root surface (RS), root diameter (D), specific root length (SRL), and root tissue density (RTD), AMF1; *Glomus* sp.2, AMF2; *G. albida*, AMF3; *G. decipiens*; and control (C); not pre-inoculated with AMF.

Salinity levels	Treatment	LRWC (%)	RFW (g)	RDW (g)	SFW (g)	SDW (g)	PH (cm)	RL (cm)	RS (cm^2^)	D (mm)	SRL (m g^−1^)	RTD (g cm^−3^)
10 (dS m^−1^)	H4 (C)	72.2c	5.3b	3.2b	4.1b	3.2b	63b	428c	39.1c	0.20b	154a	0.14b
	H4 + AMF1	73.3c	5.7b	3.5b	3.9c	2.3c	61b	434c	60.9bc	0.25a	128c	0.16b
	H4 + AMF2	91.2a	8.3a	6.0a	7.2a	5.1a	72a	845a	98.2	0.14c	142b	0.22a
	H4 + AMF3	81.5b	5.7b	3.5b	4.7b	2.6c	62b	645b	79.3ab	0.21b	150a	0.15b
	H8 (C)	69.3b	5.3c	3.0b	3.5c	2.0c	60b	491b	45.2b	0.18a	114b	0.20a
	H8 + AMF1	79.7ab	7.4b	5.7ab	4.5b	2.1bc	68ab	515b	51.9b	0.17b	110c	0.21a
	H8 + AM2	88.5a	9.8a	7.6a	6.5a	4.8a	76a	718a	75.0a	0.17b	119b	0.20a
	H8 + AM3	76.9ab	6.9bc	4.3b	4.5b	2.5b	63b	498b	54.1b	0.14c	138a	0.24a
	P6 (C)	71.1c	8.5a	5.1ab	3.6c	2.3b	56c	427c	46.8b	0.16b	137a	0.16b
	P6 + AMF1	93.3a	8.6a	6.7a	7.5a	4.7a	79a	621a	66.0a	0.16b	96c	0.24a
	P6 + AMF2	85.2b	6.1b	3.7b	4.7b	2.4b	63b	516b	52.1b	0.18a	91c	0.23a
	P6 + AMF3	84.6b	7.2ab	5.0ab	5.2b	2.4b	66b	484c	46.5b	0.16b	105b	0.22a
15 (dS m^−1^)	H4 (C)	63.2b	4.4b	2.5b	3.2b	1.5b	52b	259c	26.3b	0.17bc	120a	0.13b
	H4 + AMF1	65.2b	4.9b	2.7ab	2.9b	1.5b	54b	234c	32.6b	0.23a	115a	0.17b
	H4 + AMF2	84.6a	6.6a	4.3a	5.2a	2.9a	65a	728a	58.0a	0.15c	100b	0.25a
	H4 + AMF3	68.5b	5.2ab	3.1ab	3.2b	1.6b	54b	345b	42.0ab	0.20b	119a	0.17b
	H8 (C)	60.7c	4.1b	1.9c	3.1c	1.6c	55b	218c	28.7d	0.18b	111b	0.20b
	H8 + AMF1	67.4bc	6.7a	3.8b	4.0b	1.8b	62ab	379b	37.4c	0.23a	115b	0.17b
	H8 + AM2	81.8a	8.2a	5.8a	5.6a	4.1a	68a	581a	65.7a	0.19b	112b	0.20b
	H8 + AM3	71.3b	4.6b	2.6c	3.1c	2.1b	59b	385b	43.4b	0.15c	137a	0.28a
	P6 (C)	63.6c	4.2b	1.8c	3.1c	1.6bc	53c	244b	24.4c	0.14b	172a	0.21a
	P6 + AMF1	88.7a	7.3a	4.6a	6.4a	4.1a	71a	552a	60.1a	0.19a	85d	0.22a
	P6 + AMF2	76.5b	4.7b	2.7bc	3.2bc	1.5c	58b	379ab	37.9b	0.16a	133b	0.29a
	P6 + AMF3	74.3b	6.1ab	3.5ab	3.8b	2.0b	59b	360ab	24.4c	0.18a	122c	0.29a
20 (dS m^−1^)	H4 (C)	53.6b	4.1ab	2.3ab	2.9b	1.2b	55bc	234b	22.2c	0.18b	154a	0.20a
	H4 + AMF1	56.0b	4.0ab	2.2ab	2.7b	1.3b	53c	273b	28.4b	0.15b	153a	0.21a
	H4 + AMF2	75.7a	5.6a	3.1a	4.4a	2.7a	61a	481a	33.6a	0.11c	145b	0.29a
	H4 + AMF3	57.3b	3.4b	1.6b	2.5c	1.1b	56b	251b	33.4a	0.22a	113c	0.17b
	H8 (C)	42.8c	3.2c	1.2c	2.6c	0.8c	59b	185c	19.5c	0.17b	120bc	0.19b
	H8 + AMF1	53.5b	5.0b	2.8b	4.2b	1.6b	63ab	263bc	27.8b	0.24a	113c	0.16b
	H8 + AM2	69.7a	6.9a	4.3a	4.8a	2.9a	69a	382a	47.8a	0.19ab	115c	0.19b
	H8 + AM3	55.6b	3.0c	1.8bc	2.7c	1.7b	61ab	306ab	32.4b	0.13c	187a	0.23a
	P6 (C)	44.3c	2.8b	1.3b	2.5c	1.3b	55c	208b	19.5d	0.11c	220a	0.16b
	P6 + AMF1	70.8a	5.1a	3.4a	5.5a	3.4a	75a	442a	52.5a	0.22a	88c	0.26a
	P6 + AMF2	58.7b	2.2ab	1.8b	2.3c	1.3b	63b	304ab	34.1b	0.18ab	153b	0.17b
	P6 + AMF3	56.8b	4.6ab	2.7a	3.0b	1.4b	63b	293ab	29.4bc	0.21ab	132b	0.16b

Values followed by different letters, per salinity level and clone, are significantly different (*P* ≤ 0.05) by HSD.

Leaf relative water content (LRWC) was also significantly affected by salinity (S), AMF, eucalyptus clone (C), and the interaction of AMF × C and S × C (Table 1). Salinity reduced, but mycorrhizal plant increased LRWC. Again, eucalyptus clone H8 that was pre-inoculated with *G. albida* and clone P6 pre-inoculated with *Glomus* sp.2, showed the highest positive mycorrhizal effect (Table 3). There are several reasons why the AMF plants have a higher LRWC, (1) AMF roots have higher hydraulic conductivity at low water potential^36^; (2) AMF induce alterations to the root system^37^; (3) mycorrhizal plants have higher stomatal conductance^38^; (4) AMF accumulate solutes and improve plant osmotic adjustment^39^, and (5) improved water relation by AMF hyphae^40^.

Root length and root surface area were both significantly affected by salinity level, AMF and interaction of AMF × C. In the case of root surface area, the interaction of S × C was also significant (Table 1). Root length was significantly positively correlated with LRWC. Root diameter showed a significant negative correlation with root length, specific root length, and root tissue density (Table 4). Salinity reduced, and pre-inoculation with mycorrhiza increased, root length and root surface area (Table 3). Seedlings pre-inoculated with *Glomus* sp.2 had larger root diameter than control seedlings and seedlings pre-inoculated by both *Gigaspora* species, an effect described before^41^ and likely due to hormonal effects.

**Table 4:** Correlations between eucalyptus root architecture and leaf relative water content.

Variables2	Root length	Root diameter	Specific root length	Root tissue density	LRWC
Root length	x				
Root diameter	− 0.33**	x			
Specific root length	0.20 ns	− 0.39**	x		
Root tissue density	0.13 ns	− 0.70**	− 0.17 ns	x	
LRWC (%)	0.78**	− 0.06 ns	− 0.10 ns	0.06 ns	x

**and *ns* significant at *P* ≤ 0.01 and non-significant probability levels, respectively.

### Plant nutrient concentration

AMF, salinity, and eucalyptus clone were all significant sources of variation, and many interactions were significant as well (Table 1). Especially the interaction of AMF × S was significant for N, Na and the K/Na ratio, but not for P and K. Concentrations of N, P and K in plant shoots decreased with high salinity, while those of Na increased. The mycorrhizal effect on lowering Na concentrations was stronger than the mycorrhizal effect in increasing K concentrations; in combination, pre-inoculation with AMF increased the K/Na ratio three- to sixfold. Pre-inoculation with AMF increased leaf nutrient concentrations compared to the non-inoculated control across all salinity levels. Eucalyptus clones H4 and H8 benefitted most when pre***-***inoculated with *G. albida*, showing higher N, P, K, and lower Na concentrations than the control whereas P6 was positive when pre-inoculated with *Glomus* sp.2 (Table 5). Many studies have reported that increasing salinity levels lowered N and K concentrations, for example in pepper (*Piper nigrum* L.), olive (*Olea europaea* L.), peanut (*Arachis hypogaea* L.) and faba bean (*Vicia faba* L.)^42–45^. High concentrations of K can maintain K/Na ratio and photosynthetic rate. Higher phosphorus (P) uptake in all pre-inoculated plants is consistent with the major role of AMF in extending the depletion zone of P in the rhizosphere and increasing P uptake. Both a higher-affinity uptake system and a lower threshold concentration for absorption by AMF than by plant roots are major mechanisms of higher P uptake^46,47^.

**Table 5:** Influence of different salinity levels on nitrogen (N), phosphorus (P), potassium (K) and sodium (Na) concentrations and K/Na mass ratio in eucalyptus shoot tissue. AMF1; *Glomus* sp.2, AMF2; *G. albida*, AMF3; *G. decipiens*, and control (C); without pre-inoculation**.**

Salinity levels	Treatments	N (mg kg^−1^)	P (mg kg^−1^)	K (mg kg^−1^)	Na (mg kg^−1^)	K/Na ratio
10 (dS m^−1^)	H4 (C)	23b	1.9b	15c	38a	0.39c
	H4 + AMF1	28ab	2.2ab	26b	17b	1.52b
	H4 + AMF2	38a	3.2a	34a	11d	3.00a
	H4 + AMF3	27ab	2.7ab	25b	14c	1.74b
	H8 (C)	20c	2.0b	17c	35a	0.48c
	H8 + AMF1	29b	2.2ab	27b	26b	1.03b
	H8 + AM2	37a	2.9a	39a	23c	1.73a
	H8 + AM3	24bc	2.3ab	24b	25ab	0.94b
	P6 (C)	21b	1.4b	18c	33a	0.54c
	P6 + AMF1	30a	2.7a	36a	15c	2.37a
	P6 + AMF2	26ab	2.4a	30b	24b	1.22b
	P6 + AMF3	26ab	2.4a	31b	26b	1.18b
15 (dS m^−1^)	H4 (C)	17b	1.4b	17b	33a	0.51c
	H4 + AMF1	24a	2.0ab	18b	14b	1.27b
	H4 + AMF2	26a	2.4a	25a	12c	2.13a
	H4 + AMF3	16b	2.7ab	18b	17b	1.06b
	H8 (C)	16b	1.6b	19c	32a	0.60c
	H8 + AMF1	22a	1.8ab	30a	29a	1.05b
	H8 + AM2	24a	2.2a	26b	12b	2.14a
	H8 + AM3	22a	2.2ab	18c	15b	1.20b
	P6 (C)	17b	1.3b	12c	32a	0.37c
	P6 + AMF1	21a	2.4a	28a	20b	1.40a
	P6 + AMF2	19ab	2.1a	21b	14c	1.46a
	P6 + AMF3	21a	2.2	21b	19b	1.11b
20 (dS m^−1^)	H4 (C)	14b	1.3a	15a	33a	0.45c
	H4 + AMF1	20a	1.6a	13a	11b	1.15b
	H4 + AMF2	21a	1.9a	16a	12b	1.56a
	H4 + AMF3	20a	2.0a	15a	13b	1.14b
	H8 (C)	11b	1.2a	12b	23a	0.52c
	H8 + AMF1	18ab	1.5a	**1**9a	13b	1.52a
	H8 + AM2	19a	1.6a	17b	11b	1.50a
	H8 + AM3	18a	1.5a	11c	10b	1.06b
	P6 (C)	15b	1.1b	18b	28a	0.64b
	P6 + AMF1	18ab	2.3a	20a	12c	1.75a
	P6 + AMF2	20a	1.7ab	22a	15b	1.41a
	P6 + AMF3	18a	1.8a	14c	10c	1.44a

Values followed by different letters, per salinity level and clone, are significantly different (*P* ≤ 0.05) by HSD.

### Leaf chlorophyll concentration

**Figure 1:**
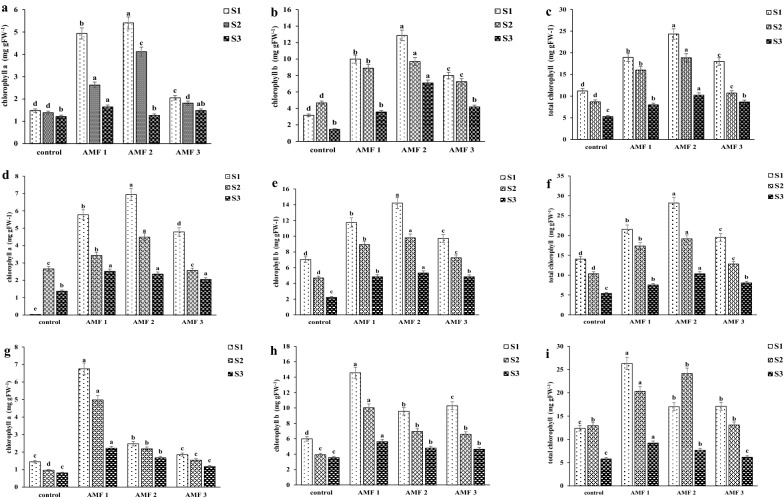
Effect of AMF pre-inoculation and salinity on leaf chlorophyll a (panels **a**,**d**,**g**); chlorophyll b (panels **b**,e,**h**) and total chlorophyll concentration (panels **c**,**f**,**i**) of eucalyptus three eucalyptus clones (H4; panels **a**–**c**), H 8: panels **d**–**f**) and P6 (panels **g**–**i**) after cultivation for 90 days. Mean values shown, in which the same letters above the bars represent no significant difference, according to HSD at *P* ≤ 0.05. Abbreviation: AMF1; *Glomus* sp.2, AMF2; *G. albida*, AMF3; *G. decipiens*, control; not pre-inoculated with AMF, S1; 10 dS m^−1^, S2; 15 dS m^−1^, S3; 20 dS m^−1^.

Leaf chlorophyll concentration, an important physiological indicator for plant photosynthetic capacity, was significantly affected by all three main factors (salinity, AMF, eucalyptus clone) and by all two-way and three-way interactions (Table 1). Salinity significantly reduced leaf chlorophyll concentration (Fig. 1) likely caused by repression of specific enzymes of the photosynthesis system and reduction of nutrient uptake such as Magnesium (Mg) and Nitrogen (N) for chlorophyll biosynthesis^48,49^. Mycorrhiza significantly increased leaf chlorophyll concentration. This result is likely due to enhanced nutrient uptake and reduced Na concentrations in the plants, resulting in overall higher photosynthetic capability^50^. In some combinations of eucalyptus clone and AMF species, there was a major effect when increasing salinity levels from 10–15 dS m^−1^, whereas in other combinations a major decline was observed only when salinity increased from 15 to 20 dS m^−1^. Due to the fact that two-way and three-way interactions were significant, other patterns were difficult to explain. Eucalyptus clones H4 and H8 pre-inoculated with *G. albida* had higher chlorophyll concentration compared to other AMF treatments, while eucalyptus clone P6 pre-inoculated with *Glomus* sp.2 had higher leaf chlorophyll concentration than the other AMF treatments.

### Leaf proline concentration

**Figure 2:**
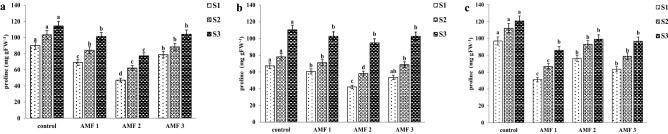
Effect of salinity level and AMF pre*-*inoculation on leaf proline concentration of eucalyptus clone H4 (**A**), H8 (**B**) and P6 (**C**) 90 days after planting. Mean values shown, in which the same letters above the bars represent no significant difference, according to HSD at *P* ≤ 0.05. Abbreviation***:*** AMF1; *Glomus* sp.2, AMF2; *G. albida*, AMF3; *G. decipiens*, control, not pre-inoculated with AMF. S1; 10 dS m^−1^, S2; 15 dS m^−1^, S3; 20 dS m^−1^.

The accumulation of free amino acid, proline-reported modifications induced by water and salt stress, and an exogenous application of proline could play an important role in enhancing plant stress tolerance^3,49^. In saline conditions, plants can accumulate proline as a protective osmolyte, maintain an osmotic balance, stabilize proteins and membranes, protect plants against free radical-induced damage, and maintain appropriate NADP^+^/NADPH ratios^51,52^. Our study resulted that leaf proline concentrations were significantly affected by all main factors (AMF, S, C) and all two-way and three-way interactions (Table 1). Proline concentrations increased with increasing salinity and were lower in AMF pre-inoculated seedlings compared with control plants, At the lowest salinity level there were significant differences between varieties, with H8 showing lowest proline concentration and H4 showing highest concentrations. With increasing salinity levels, the differences between the eucalyptus clones attenuated. Clones H4 and H8 pre-inoculated with *G. albida* and P6 pre-inoculated with *Glomus* sp.2 had significantly lower proline concentrations across all salinity levels (Fig. 2). Proline concentrations were negatively correlated with the concentrations of chlorophyll a, chlorophyll b, and total chlorophyll (Table 6). Apparently, higher nutrient uptake, LRWC, and chlorophyll content due to the mycorrhizal symbiosis constitute an alternative way to alleviate salt stress without increasing proline production. Many authors have reported that proline concentrations increased in AMF plants compared to non-AMF plants^53^, while other authors have reported greater proline accumulation in non-AMF plants than AMF plants for example, in *Ocimum basilicum* L. and *Arachis hypogaea* L^6,54^. The underlying mechanisms deserve further study.

**Table 6:** Correlations between chlorophyll a, chlorophyll b, total chlorophyll and proline concentration.

Variable	Chlorophyll a	Chlorophyll b	Total chlorophyll	proline
Chlorophyll a	x			
Chlorophyll b	0.83**	x		
Total chlorophyll	0.83**	0.92**	X	
Proline	− 0.70 **	− 0.89**	− 0.89**	x

**Significant at *P* ≤ 0.01 at probability level.

## Conclusions

Salinity reduced the growth and performance of eucalyptus seedlings due to negative effects of Na on physiological and biochemical parameters. Salinity reduced the uptake of important mineral nutrients. AMF species mitigated these negative effects by increasing the uptake of major elements (N, P, K) and the reducing uptake of Na, resulting in a much more favorable K/Na balance of pre-inoculated plants than in non-inoculated ones. Pre-inoculation with AMF also reduced plant proline concentrations, the osmoprotectant that could help non-mycorrhizal plants to alleviate salt stress. However, enhanced proline production was a less successful strategy for plant salt tolerance compared to the mycorrhizal symbiosis. Different eucalyptus clones had specific relations with certain AMF species to reduce the negative impacts of salinity on the studied physiological and biochemical parameters. Eucalyptus clones H4 and H8 pre-inoculated with *G. albida* and clone P6 pre-inoculated with *Glomus* sp.2 resulted in AMF plants that had a better growth in saline soil. Pre-inoculation with AMF therefore seems an important practice to obtain healthy eucalyptus plants in saline soils.
